# Publishing Clinical Trial Results: The Future Beckons

**DOI:** 10.1371/journal.pctr.0010031

**Published:** 2006-10-27

**Authors:** Elizabeth Wager

Search for the truth is the noblest occupation of man; its publication is a duty. —Madame de Stael (1766–1817)

Formats for reporting results from randomized clinical trials in peer-reviewed journals have remained virtually unchanged and unchallenged for the past 50 years [1]. However, a number of developments, some technological and some political, provide exciting opportunities to question whether we are using the most efficient and effective methods of publication. Here, I suggest how methods of reporting clinical trials could be improved and consider the implications for trial sponsors and medical journals.

## Why Do We Publish Clinical Trial Results?

Publication (and non-publication) of trial results can be motivated by several factors. Research reports may affect clinical practice, inform patients, influence future research, and prevent duplication of effort. Many believe there is a moral obligation to report results of all trials that involve patients [2].

Publication also has important secondary effects. These may explain existing patterns of publication and resistance to change—despite evidence that current systems are not the best. Authorship of papers in journals can establish reputations and enhance career prospects. Institutional or corporate reputations may also benefit. The productivity of academic departments is judged on their publication output, which also affects their chances of obtaining future funding. The peer-reviewed (and indexed) journal has thus become part of the process of academic appointments and promotions, which, in turn, has spawned the discipline of bibliometrics [3]. And, of course, there are financial interests: drug companies use publications to increase sales while publishers make money both directly from journal sales and from spin-offs such as reprints and advertising [4].

## Problems with Existing Publishing Models

The traditional system of publishing trials in medical journals reflects many of the limitations of paper-based systems despite the advent of electronic publishing. In general, new media and the Internet have been used to modify existing systems rather than for dramatic redesign of the architecture of the medical evidence base. For example, papers may now be accessed from journal Web sites but the format of research articles and the ways in which findings are presented remain the same. While some journals allow additional material to be displayed electronically, most still impose strict size limits on research reports and simply use Web sites as a means of accessing documents that are identical to the print version. The result is that, while there have been some major changes such as the development of “open access” journals, information remains hard (or expensive) to access, scattered across many places, and difficult to synthesize. Producing and disseminating printed material can be slow, and journal peer-review often contributes further delays [5].

Another problem with the existing system is that non-publication of negative trials and non-reporting of negative outcomes, coupled with redundant publication of positive findings, has led to systematic publication bias, which can undermine the reliability of medical evidence [2,6,7].

## Benefits of Alternative Models

One of the most important initiatives aimed at reducing publication bias is trial registration (i.e., making details of study designs publicly available at the start of a study) [8]. One potential advantage of electronic publication coupled with trial registration is that study results can be linked to the original protocol, or at least to a summary of its main features. Clear study identification, e.g., by including a trial register number, should highlight redundant publications. Secondary analyses will be easy to identify, and the risk of inadvertently including the same results more than once (which can bias systematic reviews and meta-analyses) will thus be reduced [6]. Reviewer (and reader) access to information about trial designs and planned outcomes, which was recorded at the start of each trial, will permit detection of selective reporting since reports of results can be compared with the original protocol [9,10].

Suppression of unfavourable findings will be apparent if all studies are registered at inception. But trial registration can prevent publication bias only if it is coupled with a commitment to make results from all studies available and a mechanism for implementing and policing this. Guidelines for pharmaceutical companies [11] and investigators [12] have long emphasized the importance (and some would argue moral obligation) of publishing results from all studies, regardless of their outcome. But if such guidelines are to be enforced, we need a clear definition of what is meant by publication.

## What Do We Mean by Publication?

In the era when print technology was dominant, publication was easy to define. The need for expensive equipment and expertise restricted entry into large-scale publishing activities. This was the golden age of the publisher. Within science and medicine, peer review also became established (despite limited evidence of its effectiveness and lack of agreement about its primary purpose) [13]. Until recently, publication therefore meant being printed in a peer-reviewed journal. However, a combination of new technology and increasing public calls for transparency means that different modes of publication are now being explored. A comparison of some publication methods is shown in Table 1.

**Table 1:**
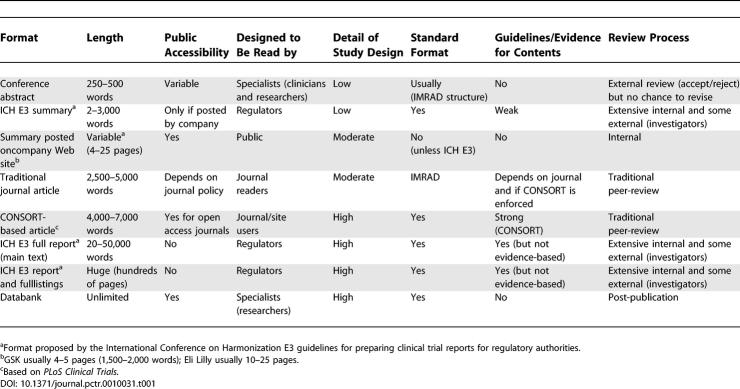
Comparison of Various Methods of Results Publication

### Results posting on Web sites.

Following legal action against companies accused of suppressing unfavourable findings, several pharmaceutical companies have announced plans to make study findings available via Web sites [14,15]. The American industry association PhRMA (the Pharmaceutical Research and Manufacturers of America) has created a Web site for this purpose (http://www.clinicalstudyresults.org).

The World Health Organization (WHO), which has been coordinating discussion about trial registries, has also discussed guidelines for the posting of results. An initial working group suggested that the summary format used for regulatory reports might be used for posting results [16]. (This summary is sometimes termed ICH E3, referring to the recommendations of the International Conference on Harmonisation, which sets reporting standards.) This format is now used by several companies on their Web sites, although critics point out that it has not been tested and no guidelines exist about the amount of detail that should be provided under each heading [17].

However, since most journals will not publish material that has already been published elsewhere, some companies have stated that results summaries will not be posted if the trial is pending publication in a peer-reviewed journal [18]. Journals have always accepted that publication of conference abstracts would not jeopardize subsequent full publication; however, summaries posted by Eli Lilly and GlaxoSmithKline range from three to 20 pages, and therefore contain considerably more detail than abstracts, so the debate continues about whether journal editors should consider such postings to be “full” publications [19]. It is ironic that medical journals, for so long the bastion of publishing research findings, may now prevent or delay other, possibly better, forms of publication.

### The role of peer-review and traditional journals.

Publication in peer-reviewed journals has, until now, been the main mechanism for disseminating findings to clinicians and researchers. Yet the evidence that peer review in medical journals acts as an effective measure of quality control is, at best, extremely limited [13]. Although they have not been through journal peer review, trial summaries prepared for regulatory reports are usually subjected to stringent quality control mechanisms and are scrutinised by both company personnel and external investigators. It could be argued that these processes are at least as good as traditional peer review, but others will argue that self-regulation by the industry is never sufficient.

Despite the fact that traditional papers are considered by many to be the “gold standard” for clinical trial publication, their effectiveness in conveying information has rarely been tested [1], they fail to provide enough detail for some users [19], and selective reporting of outcomes is common [10].

**Box 1. Reporting Results from Clinical Trials**

**What's the problem?**

- Clinical trial results are scattered and often difficult to access.
- Non-publication of unfavourable trials or negative results causes systematic publication bias.
- Standard journal articles often omit important details.
- Traditional peer-review and journal publishing delays the publication of findings.
- Enforcing commitments to publish all results are hampered by a lack of a clear definition of publication.

**What are the opportunities?**

- Trial registers/linking protocols with publications.
- Online publication on Web sites with minimal running costs.
- Data banks are a possibility for the future.

**What's the solution?**

- If all trials were registered at inception, redundant publication would be easy to spot and non-publication could be challenged.
- Links between trial protocols (or registers) and results would highlight selective reporting and post hoc analyses.
- Electronic templates for results reporting should raise standards and improve searchability.
- Results could be posted on publicly accessible Web sites at minimal cost.
- Peer-reviewed journals should concentrate on interpretation, synthesis, commentary, and discussion.

The standards for reporting randomized trials in journals have been raised by guidelines such as the CONSORT statement [20], yet there is still considerable room for improvement [21,22]. In particular, statistical errors continue to be a problem [23]. In considering the merits of various forms of publication, it would be interesting to compare journal articles with ICH E3 study reports in terms of their completeness, compliance with CONSORT, and quality of statistical analyses. It would also be interesting to compare peer review as performed by journals with the quality-control mechanisms used by companies when preparing reports—for example, in terms of their ability to detect errors.

Journal articles using the traditional IMRAD format (i.e., Introduction, Methods, Results, And Discussion) combine objective study findings with subjective interpretation. While evidence of many other supposed benefits of peer review is weak, its ability to “tone down” conclusions and remove unfounded claims has been clearly shown [13]. But peer-review has a poor record in detecting incorrect or fabricated data [5]. If the functions of reporting results and discussing their implications are separated, one might argue that peer review is more helpful for the latter than the former. A new model might therefore be for investigators or sponsors to make results available on publicly accessible Web sites using standard templates and for journals to add value by publishing peer-reviewed commentary and synthesis.

This model might also help resolve the ethical arguments about access to research. Funders, researchers, and readers increasingly demand access to clinical trial results for free, yet producing peer-reviewed journals is a costly business [24]. If results were freely accessibly from publicly funded Web sites, then the costs and delays involved with peer review would not affect the primary dissemination of results. Publishers and editors could justify journal access charges by producing lively and informative reviews and critiques of the latest findings. Journals (or electronic media) could also specialize in providing information and interpretation for different audiences, for example, to meet the different needs of researchers, clinicians, and patients.

## Looking Further into the Future

While free text may serve readers well, it is not an efficient electronic storage medium. The most powerful, and some would argue the most efficient and transparent, method of making results available would be to provide raw (but anonymized) datasets for public scrutiny. It is now technically possible to store results in a computable databank as in the Trial Bank Project (http://rctbank.ucsf.edu/). Based on such research, a Global Trial Bank has recently been established and aims to be “the most advanced computable repository of trial protocol and results information to promote biomedical discovery as well as transparency and accountability in clinical trial research” [16]. Companies already provide such data to the US Food and Drug Administration, proving that this is technically possible and that standard data transfer protocols are available.

However, adoption of such an initiative requires not only technical feasibility but also political and commercial acceptability. Individuals, academic institutions, and commercial companies usually consider data as a precious possession, not something to be shared with potential rivals. Many researchers are concerned that public access to raw data could encourage inappropriate analyses and misinterpretation. However, Senn has suggested that providing computable data might become “the ultimate test and promoter of statistical quality” [25]. He argues that “every statistician will have to prepare reports knowing that they will be scrutinised in the finest detail by a frequently hostile posterity”. This, surely, is the ultimate form of peer review. However, although databanks offer exciting possibilities, researchers' reluctance to expose their raw data to the world may hold back change.

## Problems Ahead

Publicly accessible, searchable electronic results dissemination coupled with trial registers could solve many of the problems that limit the current system of research reporting via peer-reviewed journals. However, such a new system could also create problems of its own.

For a start, trial registers and publication vehicles need to be funded. Currently, some registers and Web sites charge an administration fee for entries. This may be a barrier for unfunded research and projects from the developing world. It is therefore vital that sustainable funding systems are devised, and these must allow inclusive policies for trial registration and results dissemination.

Researchers will need to be equipped to produce reports or data in the required format. This might be facilitated by open-access software and protocols for data transfer, but human effort will also be required for education and support.

The proposed system of posting results is largely self-policing and relies to a greater extent on internal systems of quality control (within companies or academic institutions) than traditional peer-reviewed publication. Performing trials and handling data according to Good Clinical Practice requires considerable expertise and resources. This is currently reinforced by oversight from regulatory bodies (e.g., random audits) in the case of the pharmaceutical industry. The new model of publication might require similar policing systems, especially for non-industry-funded studies, and such systems would require considerable resources.

Editors and publishers may be concerned about what will happen to their journals if they are no longer the primary vehicle for publishing clinical trial results. Some medical editors have already considered and even prophesied this scenario [26–28]. Under the new system, successful journals will be those brave enough to focus on other functions. These could include secondary publications providing synthesis, commentary, opinion, and interpretation, along with other roles such as stimulating debate, campaigning for change, educating, and even entertaining their readers [26]. Freed from the responsibility of publishing clinical trial results, journals might also develop new and more effective models of peer review.

The current system of academic credits and research assessment based on authorship of peer-reviewed articles would need to be changed. Entries on trial registers and results databases are currently anonymous. If electronic posting becomes the primary (or, for some studies, the sole) method of publication, a fair and transparent system for acknowledging contributors will need to be devised. This should be viewed as an opportunity to replace traditional journal authorship systems, which are open to abuse [29]. Better methods of assessing academic performance than relying on journal impact factors should also be developed [30]. Use of study identifiers presents possibilities to develop more accurate citation measures relating to the original study rather than its publication in a journal—for example, by measuring the number of times that a study is re-analysed, synthesized, or referred to in journals.

## Conclusions

We are at the threshold of a new era of reporting clinical trial results. We need to develop an evidence-based system to ensure that data storage and dissemination technology are harnessed to produce the best outcome for all users. Given the huge sums spent on medical research, it is a scandal that we have invested so little in determining the most effective and efficient ways of disseminating findings. I urge funders, especially non-commercial ones such as the Wellcome Trust, Medical Research Council (in the UK), and National Institutes for Health (in the US), to rise to this challenge. We also need to consider the best funding models for such systems that would optimize access to findings without creating barriers to non-commercially funded research. Changes in research reporting systems will have implications for the future of medical journals and for current systems of measuring research productivity. Given the established position of peer-reviewed journals, there is likely to be a gradual transition from the current model. This transition may be accelerated by influencers such as journal editors (if they are brave enough to embrace the change rather than resisting it), regulators, and legislators. With wide consultation and cooperation from all key players (including clinicians, drug companies, and bioinformatics experts), we could produce a system that greatly enhances the efficiency, transparency, and power of the medical knowledge base. This is an opportunity we must not miss.
